# Glyoxalase I disruption and external carbonyl stress impair mitochondrial function in human induced pluripotent stem cells and derived neurons

**DOI:** 10.1038/s41398-021-01392-w

**Published:** 2021-05-08

**Authors:** Tomonori Hara, Manabu Toyoshima, Yasuko Hisano, Shabeesh Balan, Yoshimi Iwayama, Harumi Aono, Yushi Futamura, Hiroyuki Osada, Yuji Owada, Takeo Yoshikawa

**Affiliations:** 1 Laboratory of Molecular Psychiatry, RIKEN Center for Brain Science, Wako, Saitama 351-0198 Japan; 2 Department of Organ Anatomy, Tohoku University Graduate School of Medicine, Sendai, Miyagi 980-8575 Japan; 3 Neuroscience Research Laboratory, Institute of Mental Health and Neurosciences (IMHANS), Kozhikode, Kerala 673008 India; 4 Support Unit for Bio-Material Analysis, Research Division, RIKEN Center for Brain Science, Wako, Saitama 351-0198 Japan; 5 Chemical Biology Research Group, RIKEN Center for Sustainable Resource Science, Wako, Saitama 351-0198 Japan

**Keywords:** Molecular neuroscience, Physiology

## Abstract

Carbonyl stress, a specific form of oxidative stress, is reported to be involved in the pathophysiology of schizophrenia; however, little is known regarding the underlying mechanism. Here, we found that disruption of *GLO1*, the gene encoding a major catabolic enzyme scavenging the carbonyl group, increases vulnerability to external carbonyl stress, leading to abnormal phenotypes in human induced pluripotent stem cells (hiPSCs). The viability of *GLO1* knockout (KO)-hiPSCs decreased and activity of caspase-3 was increased upon addition of methylglyoxal (MGO), a reactive carbonyl compound. In the *GLO1* KO-hiPSC-derived neurons, MGO administration impaired neurite extension and cell migration. Further, accumulation of methylglyoxal-derived hydroimidazolone (MG-H1; a derivative of MGO)-modified proteins was detected in isolated mitochondria. Mitochondrial dysfunction, including diminished membrane potential and dampened respiratory function, was observed in the *GLO1* KO-hiPSCs and derived neurons after addition of MGO and hence might be the mechanism underlying the effects of carbonyl stress. The susceptibility to MGO was partially rescued by the administration of pyridoxamine, a carbonyl scavenger. Our observations can be used for designing an intervention strategy for diseases, particularly those induced by enhanced carbonyl stress or oxidative stress.

## Introduction

Schizophrenia is a debilitating psychiatric disorder, which is etiologically heterogeneous^1–3^. Recently, carbonyl stress, a type of oxidative stress, has been reported to be elevated in a subset of cases of schizophrenia^4,5^. Carbonyl stress is a condition associated with prolific accumulation of advanced glycation end products (AGEs)^6^. AGEs are damaged proteins, amino acid residues of which are irreversibly modified by reactive carbonyl compounds (RCOs)^7,8^. This reaction is mediated by the close interaction between free radical oxidation and glycation^9^. AGE accumulation is known to elicit activation of inflammatory signaling via the receptors for AGEs^10,11^.

**Fig. 1:**
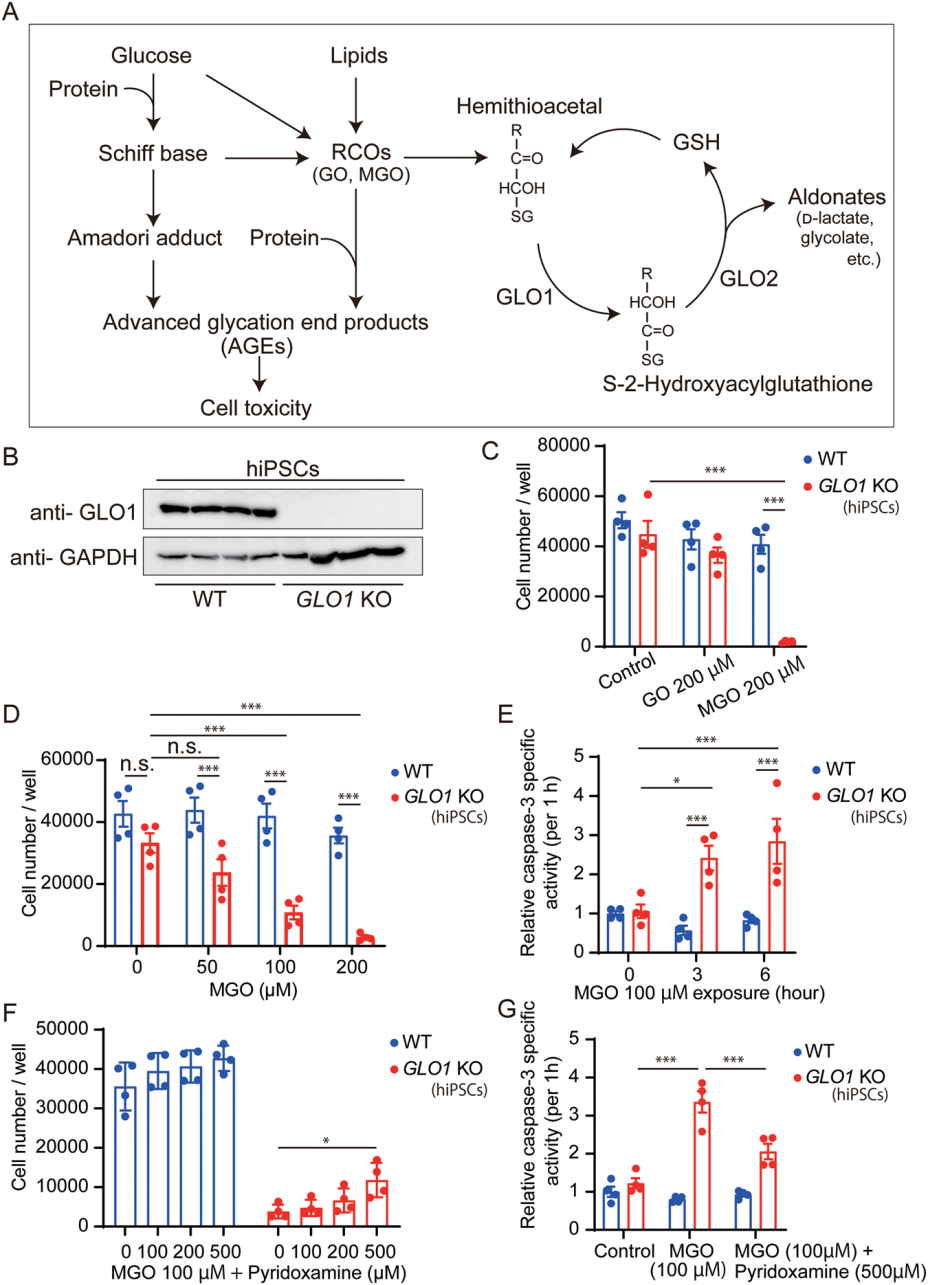
Cell viability and apoptosis in human *GLO1* KO-hiPSCs after treatment with reactive carbonyl compounds. **A** Schematic presentation of the biosynthesis of AGEs (left part) and glyoxalase cycle (right part). Reactive carbonyl compounds (RCOs) are produced from glucose and lipids to form AGEs. Accumulated AGEs induce cell toxicity. Glyoxalase I (GLO1) plays a pivotal role in removing RCOs via the glyoxalase cycle. **B** Western blot of WT- and *GLO1* KO-hiPSCs using an antibody against GLO1. **C** Cell viability analysis in WT- and *GLO1* KO-hiPSCs after treatment with 200 µM GO and 200 µM MGO for 24 h. **D** Analysis of the viability of cells treated with 0, 50, 100, and 200 µM MGO for 24 h. *GLO1* KO-hiPSCs showed reduction in cell viability in a concentration-dependent manner. **E** Analysis of apoptosis in WT- and *GLO1* KO-hiPSCs after exposure to 100 µM MGO for 0, 3, and 6 h. Treated *GLO1* KO-hiPSCs showed increased caspase activity in a time-dependent manner. **F** Rescue assay for cell viability after treatment with 100 µM MGO mediated by co-incubation with pyridoxamine (0, 100, 200, and 500 µM) for 24 h. **G** Rescue assay for apoptosis after exposure to 100 µM MGO mediated by co-incubation with 500 µM pyridoxamine for 6 h. Data represent mean ± SEM. *P < 0.05, **P < 0.01, ***P < 0.001; two-way ANOVA, followed by Tukey’s multiple comparison test.

Organisms possess an anti-glycation defense mechanism for eliminating RCOs and suppressing the accumulation of AGEs (Fig. 1A)^8,12,13^. This defense mechanism, named “glyoxalase cycle”, converts RCOs to harmless aldonates, including d-lactate and glycolate^14^. The major enzymatic components of this system are glyoxalase I (GLO1) and glyoxalase II (GLO2)^13,15^. Among them, GLO1 is the rate-limiting enzyme^12,15^, and inhibition of GLO1 critically increases the accumulation of RCOs^16–18^. Notably, downregulated expression of *GLO1* has been reported in a wide range of neuropsychiatric disorders, including autism spectrum disorder, major depressive disorder, depressive state of bipolar disorder, and Alzheimer’s disease^19–22^. But the molecular mechanism of *GLO1* deficiency in pathogenesis of psychiatric disorders including schizophrenia remains largely unknown.

In this study, we aimed to investigate the effects of varying the load of carbonyl stress in neurodevelopment with regard to schizophrenia pathophysiology^23–25^ using *GLO1*-knockout (KO) human induced-pluripotent stem cells (hiPSCs) and *GLO1*-KO hiPSC-derived neurons.

## Materials and methods

### Establishment of hiPSCs and deletion of *GLO1*

We established hiPSCs from a healthy volunteer (47-year-old male, named THC-10). T cells from peripheral blood were cultured on dishes coated with anti-human CD3 antibody (1:100, BD, Franklin Lakes, NJ, USA) in KBM502 medium (Kohjin Bio, Saitama, Japan). We confirmed that the subject did not harbor any missense variants of the genes relevant for the carbonyl stress pathway, namely, *GLO1*, *GLO2*, and *AGER* (encoding AGE specific receptor), as described previously^26^. T cells were reprogrammed to hiPSCs using CytoTune^®^-iPS 2.0 (ID Pharma, Tsukuba, Japan) according to the manufacturer’s instructions. The hiPSCs were maintained under feeder-free conditions using the Cellartis^®^ DEF-CS™ 500 culture system (Takara Bio, Shiga, Japan). *GLO1* deletion was generated via genome editing using the CRISPR-Cas9 system. Briefly, cultured hiPSCs were transduced with single guide RNA (5′-CTTGGTACTGGGGTCCGCGTCGG-3′), Guide-it Recombinant Cas9 protein (Takara Bio), and puromycin-resistant vector pDonor-D4 (GeneCopoeia, Rockville, MD, USA) via electroporation (Neon^®^ Transfection system, Thermo Fisher, Waltham, MA, USA). After 24 h, puromycin (Nacalai Tesque, Kyoto, Japan) selection was performed for 24 h. Four wild type (WT) and four *GLO1* KO clones were established via single-cell cloning (Fig. S1). The absence of GLO1 at the protein level was confirmed via Western blotting (Fig. 1B) using anti-rabbit GLO1 antibody (Lab-made^27^). A web-based tool, COSMID (https://crispr.bme.gatech.edu), was used to identify off-target sites, and two possible sites on chromosomes 5 and 7 were identified^28^. The genomic integrity of these regions was confirmed using the Guide-it mutation detection kit (Takara Bio). All tested clones were negative for mycoplasma. All experimental procedures were approved by the ethics committee of RIKEN (approval no. Wako 3 2019-2). All methods were conducted according to the principles set out in the WMA Declaration of Helsinki and the NIH Belmont Report. The subject provided informed and written consent for participation in the study.

### Induction of carbonyl stress

Reactive carbonyl compounds were administered extrinsically to the culture medium. Cells were incubated with a major RCO methylglyoxal (MGO; Sigma Aldrich, MO, USA) or a less reactive RCO^29^ glyoxal (GO; FujiFilm Wako Pure Chemical, Osaka, Japan) for 24 h, unless otherwise noted, before performing the experiments according to previous reports^30–32^.

### Cell viability assay

Following incubation of hiPSCs with reactive carbonyl compounds for 24 h, the medium was removed and the cells were stained with Hoechst 33342 (1:2000, Thermo Fisher) in Dulbecco’s phosphate-buffered saline (DPBS). After 5 min of incubation, the staining solution was replaced, and the cells were washed with DPBS. Images were acquired via tile scanning using the Axio Observer (Zeiss, Jena, Germany). Cell number was automatically counted using the Zen software (Zeiss). We performed rescue experiments using pyridoxamine (Sigma Aldrich)^33^. For hiPSC-derived neurons, cell viability was manually analyzed using the trypan blue exclusion test^34^. Briefly, the cell suspension was obtained from the cells treated with MGO for 24 h. Suspensions were gently mixed with 0.4% (w/v) trypan blue solution to detect viable cells. The number of live cells was counted using a hemocytometer (Wakenbtech, Kyoto, Japan).

### Detection of caspase-3 activity

Apoptosis was evaluated using the APOPCYTO caspase-3 fluorometric assay kit (Medical and Biological Laboratories, Nagoya, Japan) according to the manufacturer’s instructions. Briefly, whole cell lysates were extracted after MGO treatment (0−6 h, 100 µM). These lysates were incubated with DEVD-AMC (7-amino-4-methylcoumarin) in a 96-well microplate for 60 min. DEVD-AMC is a synthetic substrate labeled with four amino acid sequences, which can be cleaved by active caspase-3 to generate free fluorescent AMC. The fluorescence was analyzed using a Varioskan microplate reader (Thermo Fisher). Data were normalized to protein levels determined using the Pierce 660 nm protein assay kit (Thermo Fisher).

### Differentiation of hiPSCs to neuronal cell lineage

Neuronal induction was performed as previously described with slight modifications^27,35,36^. Feeder-free hiPSCs were treated with 2 µM SB431542 (Sigma Aldrich), 3 µM CHIR99021 (FujiFilm Wako Pure Chemical), and 3 µM dorsomorphin (FujiFilm Wako Pure Chemical) in DEF-CS for 5−7 days. Treated hiPSCs were dissociated into single cells using TrypLE Select (Thermo Fisher). Single cells were plated at a density of 1 × 10^4^ cells/mL in uncoated T75 easy flasks (Thermo Fisher) in media hormone mix (MHM) supplemented with 2% B27 supplement (Thermo Fisher), 20 ng/mL bFGF (Peprotech, Cranbury, NJ, USA), 10 ng/mL hLIF (Nacalai Tesque), 10 nM Y-27632 (FujiFilm Wako Pure Chemical), 2 µM SB431542, and 3 µM CHIR99021. The flasks were incubated in a hypoxic and humidified atmosphere (4% O_2_ and 5% CO_2_) for 7−10 days. The medium was changed after 5−7 days. Neurospheres, which mainly consist neural stem/progenitor cells^35^, were formed with passage of time and had been dissociated into single cells after treatment with TrypLE Select^27,35^. The digestion was quenched with a 0.02% trypsin inhibitor (Sigma Aldrich). The cell suspension was plated onto vessels coated with poly-l-ornithine (15 µg/mL, Sigma Aldrich) and laminin (1:100, Thermo Fisher), and cultured for 3 days in MHM supplemented with 2% B27 supplement, 20 ng/mL BDNF (Peprotech), 10 ng/mL GDNF (Peprotech), 400 µM dibutyryl cAMP (Sigma Aldrich), 200 µM ascorbic acid (Sigma Aldrich), and 1% Cultureone supplement (Thermo Fisher).

### Neurite outgrowth assay

Approximately 20−30 neurospheres were allowed to adhere to coated 35 mm glass bottom dishes (Matsunami glass, Osaka, Japan) containing neural induction medium as stated above. For administering carbonyl stress, 50 µM MGO was added to the medium. The cells were fixed for immunocytochemistry after 48 h incubation^35^. Images were acquired using an Axio Observer (Zeiss) and analyzed using the Zen software (Zeiss). The average neurite length was measured from the edge of the cluster to the most distant end point (10 neurons per neurosphere and 10 neurospheres per given condition). The average migration distance was measured from the edge of the cluster to the nucleus of the most distant neurons (10 neurons per neurosphere and 10 neurospheres per condition).

### Imaging of mitochondrial membrane potential

Mitochondrial membrane potential was evaluated through JC-1 staining using the JC-1 MitoMP detection kit (Dojindo Laboratories, Kumamoto, Japan). JC-1 changes the fluorescent color from green to red depending on the mitochondrial membrane potential. Reduction in red (healthy mitochondria, 530 nm)/green (unhealthy mitochondria, 590 nm) fluorescence intensity ratio of JC-1 indicates depolarization of mitochondria due to conversion of red fluorescent JC-1 aggregates to green fluorescent JC-1 monomers^37^.

Cells were incubated with JC-1 solution (final concentration: 2 µmol/L) at 37 °C for 30 min after 24 h incubation with 50 µM MGO in a black/clear bottom microplate (Corning, NY, USA). The wells were washed twice with Hank’s balanced salt solution (HBSS). Then, the medium was replaced with an imaging buffer solution attached to the kit. JC-1 fluorescence was quantified using a Varioskan microplate reader (green: 485 nm (Ex), 535 nm (Em); red: 535 nm (Ex), 595 nm (Em)). Fluorescent images were generated using an Axio Observer microscope.

### ATP assay

Intracellular ATP was measured using the Cellno ATP assay reagent (TOYO B-NET, Tokyo, Japan). Briefly, cells were prepared in a white/clear bottom microplate (Thermo Fisher) and treated with 50 µM MGO for 24 h. The next day, 100 µL of assay reagent was added, and the plate was gently shaken for 1 min. After 10 min of incubation, luminescence was measured using a Varioskan microplate reader. Data were normalized to protein levels determined using the micro bicinchoninic acid (BCA) protein assay kit.

### Determination of oxygen consumption rate (OCR)

Mitochondrial OCR was measured using a Seahorse XFe96 analyzer (Agilent Technologies, Santa Clara, CA, USA) as previously described with slight modifications^38^. Briefly, cells were plated in Seahorse XFe96 microplates (Agilent Technologies) and treated with 50 µM MGO for 24 h. On the next day, the cells were pre-incubated in assay medium (Agilent Technologies) supplemented with 25 mM glucose, 4 mM glutamine, and 1 mM sodium pyruvate at 37 °C for 1 h in a CO_2_-free incubator. Next, the Seahorse XF Mito stress test was performed following sequential injections of oligomycin A (1 μM) from port A, Carbonyl cyanide 4-(trifluoromethoxy)phenylhydrazone (FCCP) (0.5 μM for hiPSCs and 1 μM for neurons^39^) from port B, and antimycin A (1 μM) plus rotenone (1 μM) from port C. Data were normalized to protein levels determined using the micro BCA protein assay kit.

### Immunocytochemistry

For immunocytochemistry, cells were fixed with 4% paraformaldehyde in DPBS. The fixed cells were permeabilized with 0.2% Triton X-100 in DPBS and blocked with 2% skim milk. The cells were incubated with mouse anti methylglyoxal-derived hydroimidazolone (MG-H1; 1:20; Cell Biolabs, San Diego, CA, USA), mouse anti-β-III tubulin (1:500; Billerica, Merck Millipore, MA, USA), and rabbit anti-NeuN antibodies (1:300; Abcam, Cambridge, UK), followed by incubation with secondary antibodies: goat anti-mouse Alexa 488, goat anti-mouse Alexa 594, and goat anti-rabbit Alexa Fluor 488 (1:500; Thermo Fisher, for all). 4′,6-Diamidino-2-phenylindole (1:1000; Roche, Basel, Switzerland) was added as a nuclear marker. Mitotracker Red CMXRos (Thermo Fisher) was used for mitochondrial labeling. After washing with DPBS, the samples were mounted in PermaFluor (Thermo Fisher) or immediately observed using Axio Observer without mounting. Images were analyzed using the Zen software.

### Western blotting of isolated mitochondria

Fresh mitochondria were isolated from hiPSCs or neurons using the Qproteome mitochondria isolation kit (Qiagen, Hilden, Germany). Mitochondria were completely dissolved in ice-cold radioimmunoprecipitation assay buffer containing a protease inhibitor cocktail (Sigma Aldrich) and phenylmethanesulfonyl fluoride (Cell Signaling, Beverly, MA, USA) as an inhibitor of serine proteases. Protein samples were loaded onto 10% sodium dodecyl sulfate polyacrylamide gels and separated via electrophoresis. Subsequently, the proteins were transferred onto polyvinylidene difluoride membranes (Merck Millipore). The membranes were blocked with 3% skim milk in Tween Tris Buffered Saline (TTBS) and incubated overnight with either mouse anti-MG-H1 (1:1000; Cell Biolabs), mouse anti-VDAC1 (1:1000; Abcam), rabbit anti-HSP60 (1:20000; Abcam) or goat anti-GAPDH antibody (1:1000; Santa Cruz Biotechnology, Santa Cruz, CA, USA) at 4 °C. After primary antibody incubation, the membranes were washed with TTBS and incubated with the following secondary antibodies for 1 h at room temperature: horse radish peroxidase (HRP)-conjugated anti-mouse IgG, HRP-conjugated anti-rabbit IgG, or HRP-conjugated anti-goat IgG (1:3000; GE Healthcare, Little Chalfont, UK, for all). Signals were detected using an Immobilon western chemiluminescent HRP substrate (Merck Millipore) and recorded with FUSION Solo S (Vilber-Lourmat, Collégien, France). Band intensity was analyzed using the ImageJ software (version 1.50i).

### Statistical analysis

Statistical comparisons were performed using unpaired *t*-test (two tails) or two-way analysis of variance (ANOVA), followed by Tukey’s post hoc test using Prism 9 (GraphPad, San Diego, CA, USA). *P*-values less than 0.05 were considered significant. Data are presented as mean ± standard error of the mean (SEM).

## Results

### *GLO1* KO-hiPSCs showed increased vulnerability to external carbonyl stress with elevation in apoptosis signaling, which was partially rescued by pyridoxamine

To examine the cytotoxicity induced by enhanced carbonyl stress, we evaluated the viabilities of WT and *GLO1* KO-hiPSCs. The WT and *GLO1* KO-hiPSCs showed almost similar viability under normal culture conditions (Fig. 1C). However, under enhanced carbonyl stress induced by adding 200 µM methylglyoxal (MGO) in the culture medium, the *GLO1* KO-hiPSCs showed a drastic decrease in cell number after 24 h, whereas the WT hiPSCs did not show any significant decrease. The addition of 200 µM glyoxal (GO) did not affect the viability of WT and *GLO1* KO cells (Fig. 1C). To determine whether lower concentrations of MGO can affect cell viability, we tested a range of MGO concentrations (0, 50, 100, and 200 µM) for 24 h. The toxicity of MGO toward *GLO1*-hiPSCs was dose-dependent, and the effect of MGO was evident at ≥50 µM (WT vs. *GLO1* KO) or at ≥100 μM (*GLO1* KO under no MGO vs. *GLO1* KO under MGO) (Fig. 1D). These results suggested that *GLO1* KO is sensitive to extrinsic carbonyl stress.

To investigate the molecular mechanism underlying the cytotoxic effects of MGO, we evaluated the apoptosis signaling pathways. MGO (100 µM) was administered at different time points (0, 3, and 6 h). Upon MGO treatment, the *GLO1* KO-hiPSCs showed elevated apoptotic signaling, as indicated by the increase in caspase-3 activity, in a time-dependent manner (Fig. 1E).

Notably, 500 μM of pyridoxamine treatment significantly, but partially, rescued the cytotoxic damages induced by MGO (100 µM, 24 h) in *GLO1* KO-hiPSCs (Fig. 1F). Furthermore, pyridoxamine (500 µM) reversed the elevation of apoptotic signaling elicited by the addition of MGO (100 µM) in the *GLO1* KO-hiPSCs (Fig. 1G).

### Carbonyl stress reduced neuronal cell viability and impaired neurite extension and migration

**Fig. 2:**
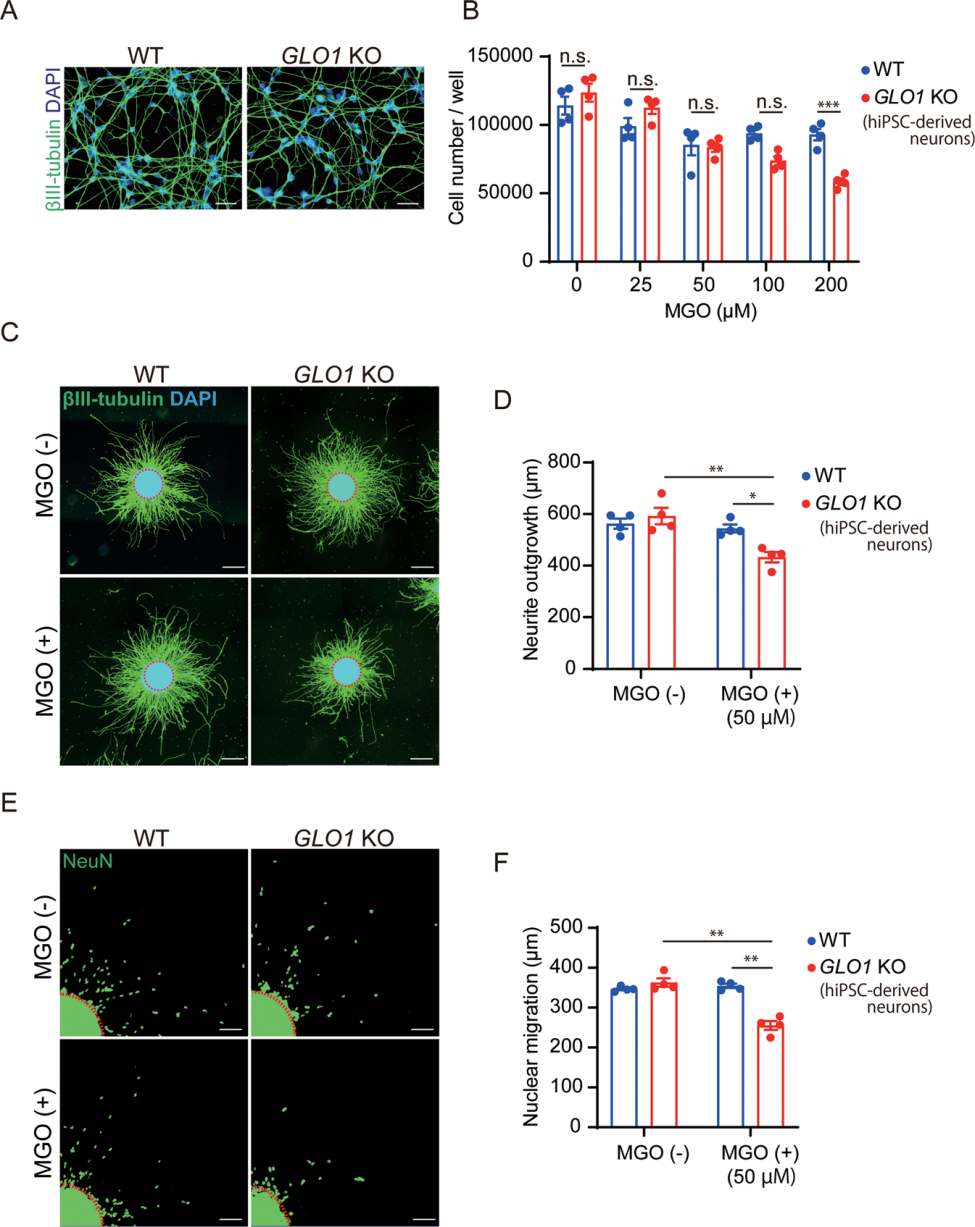
Characterization of WT- and *GLO1* KO hiPSC-derived neurons after treatment with MGO. **A** Representative immunofluorescent images of neurons derived from WT- and *GLO1* KO-hiPSCs (scale bar: 50 µm). **B** Cell viability analysis in WT and *GLO1* KO neurons after treatment with 0, 25, 50, 100, and 200 µM MGO for 24 h. Cell numbers were similar between WT and *GLO1* KO after treatment with 0, 25, 50, and 100 µM MGO. The number of *GLO1* KO neurons were significantly lower than that of WT neurons after treatment with 200 µM MGO. **C**, **D** Neurite outgrowth analysis in WT and *GLO1* KO neurons after treatment with 50 µM MGO for 48 h. Dashed line in **C**: demarcation of clustered neuronal cell bodies; scale bar: 200 µm. Neurite lengths were significantly lower in *GLO1* KO neurons after treatment with 50 µM MGO. **E**, **F** Neuronal migration analysis in WT and *GLO1* KO neurons after treatment with 50 µM MGO for 48 h. Dashed line in **D**: demarcation of clustered neuronal cell bodies; scale bar: 100 µm. Neuronal migrations were significantly lower in *GLO1* KO neurons treated with 50 µM MGO. Data represent mean ± SEM. *P < 0.05, **P < 0.01, ***P < 0.001***; two-way ANOVA, followed by Tukey’s multiple comparison test.

To assess the effects of carbonyl stress on neurodevelopment, we prepared neuronal cells that were differentiated from the neurospheres. Immunocytochemistry analysis showed that more than 95% of the differentiated cells were positive for the neuronal marker βIII-tubulin in both WT and *GLO1* KO cells and that their morphologies did not differ (Fig. 2A). The total number of neuronal cells (neurons) differentiated from the *GLO1* KO-hiPSCs was similar to that from the WT hiPSCs under normal conditions. However, the number of *GLO1* KO-hiPSC-derived neurons was significantly lower than that obtained from the WT after treatment with 200 μM MGO (24 h) (Fig. 2B). These results showed that mild carbonyl stress (*GLO1* KO alone) does not affect the viability of derived neurons, although severe stress (*GLO1* KO plus 200 μM of MGO) can dampen cell viability in derived neurons, suggesting that *GLO1* KO neurons are sensitive to extrinsic carbonyl stress.

Next, to analyze neurite extension, we prepared another type of βIII-tubulin-positive neurons, which were differentiated from the neurospheres that had been allowed to adhere to glass bottom dishes. The neurites were visualized using an anti-βIII-tubulin antibody (Fig. 2C). Under normal conditions, the neurite lengths of the WT and *GLO1* KO-derived neurons were similar (Fig. 2D). Then, we determined the biological effects of minimal MGO addition (50 μM, 48 h) on the *GLO1* KO-hiPSCs (Fig. 1D). After MGO addition, neurite lengths were significantly shorter in the *GLO1* KO-derived neurons (432 ± 20 µm) than in the non-treated *GLO1* KO-derived (592 ± 32 µm) and MGO-treated WT-derived neurons (545 ± 15 µm) (Fig. 2D). Cell nuclei were visualized using anti-NeuN antibody (Fig. 2E). The neuronal migration distance after MGO addition (50 µM, 24 h) in the *GLO1* KO-derived neurons (256 ± 11 µm) was significantly lower than that in the non-treated *GLO1* KO-derived (362 ± 11 µm) and MGO-treated WT-derived neurons (354 ± 5.3 µm) (Fig. 2F). These results showed that some extent of carbonyl stress (*GLO1* KO plus 50 μM of MGO) can impair neurite extension and neuronal migration.

### Enhanced carbonyl stress elicited mitochondrial dysfunction in *GLO1* KO-hiPSCs

**Fig. 3:**
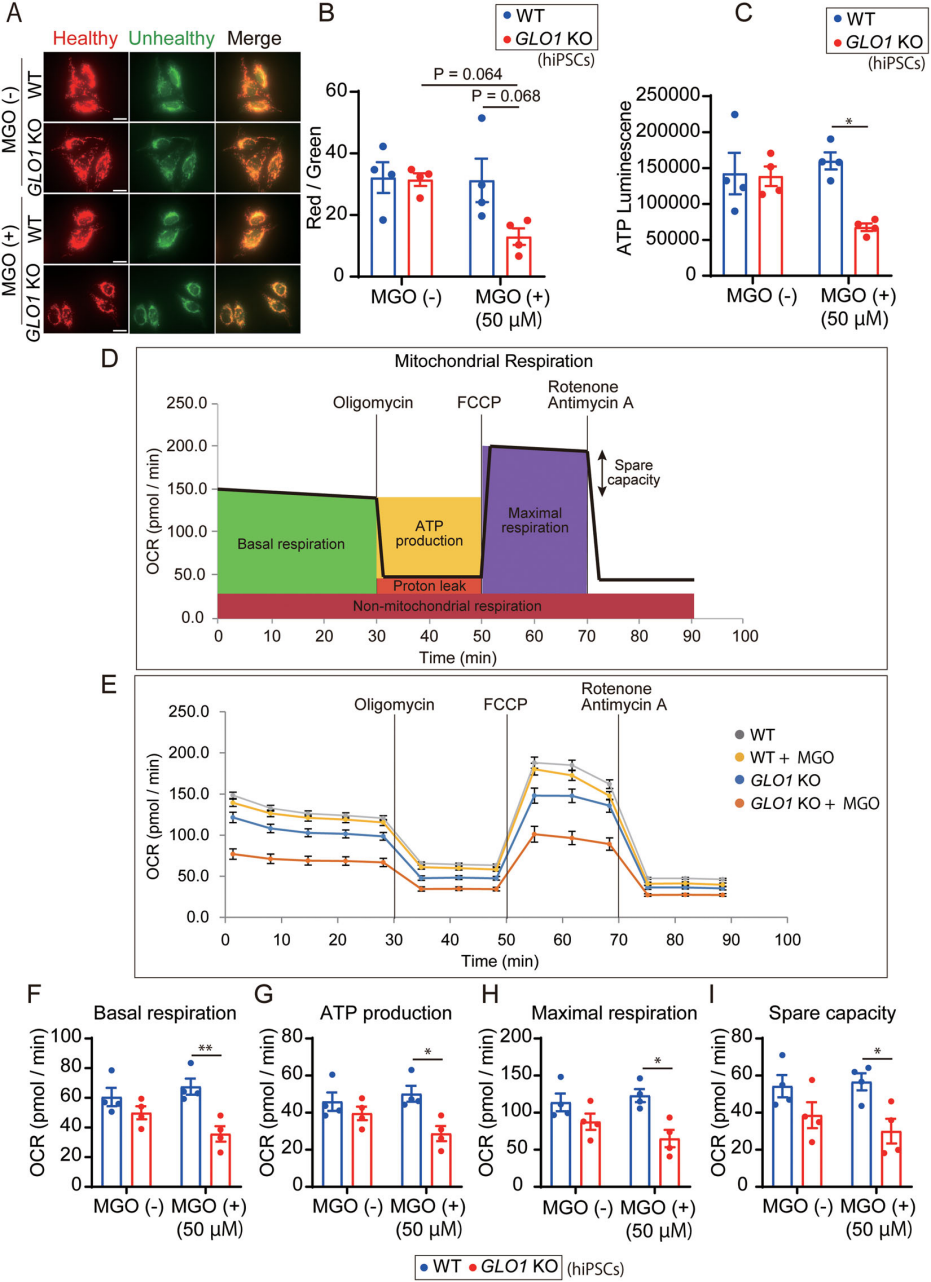
Evaluation of mitochondrial function in WT- and *GLO1* KO-hiPSCs after treatment with MGO. **A**, **B** JC-1 staining of WT- and *GLO1* KO-hiPSCs after treatment with 50 µM MGO for 24 h. Scale bar: 50 µm. **C** Measurement of intracellular ATP contents in WT- and *GLO1* KO-hiPSCs treated with 50 µM MGO for 24 h. Data were normalized to protein concentration. **D** Profile of mitochondrial respiration analysis using Seahorse extracellular flux analyzer. Oligomycin, FCCP, and rotenone + antimycin A were serially administered. OCR oxygen consumption rate. **E** Plot of OCR in WT- and *GLO1* KO-hiPSCs versus measurement time. MGO, treatment with 50 µM MGO for 24 h. Data were normalized to protein concentration. **F**−**I** Quantification of basal respiration (**F**), ATP production (**G**), maximal respiration (**H**), and spare capacity (**I**). *GLO1* KO-hiPSCs showed significant reduction in respiratory function after treatment with 50 µM MGO. Data represent mean ± SEM. *P < 0.05, **P < 0.01; two-way ANOVA, followed by Tukey’s multiple comparison test.

As carbonyl stress increased caspase-3 activities (Fig. 1E) and as mitochondria are known to play a pivotal role in apoptotic signaling^40^ and normal neuronal development^41^, we investigated the effects of enhanced carbonyl stress on mitochondrial integrity based on the fluorescence intensity ratio of JC-1. We observed that the fluorescence ratios were similar between the WT and *GLO1* KO-hiPSCs under normal conditions, although the ratios for the *GLO1* KO-hiPSCs showed a decreasing trend after MGO addition (50 µM, 24 h) compared to those under normal condition (*P* = 0.064) or those for MGO-treated WT hiPSCs (*P* = 0.068) (Fig. 3A, B). In the ATP assay, MGO treatment (50 µM, 24 h) significantly decreased the intracellular ATP content in the *GLO1* KO-hiPSCs when compared to that in the WT-hiPSCs, whereas the treatment did not affect ATP content in the WT-hiPSCs (Fig. 3C).

Next, we performed a comprehensive mitochondrial respiration analysis by measuring the oxygen consumption rate (OCR) based on four parameters (basal respiration, ATP production, maximal respiration, and spare capacity; Fig. 3D). MGO treatment (50 µM, 24 h) significantly decreased mitochondrial basal respiration, ATP production, maximal respiration, and spare capacity in the *GLO1* KO-hiPSCs (Fig. 3E−I), whereas no significant effects were observed in the WT hiPSCs. These results showed that a certain magnitude of carbonyl stress (*GLO1* KO plus 50 µM of MGO) can impair overall mitochondrial respiratory functions.

To examine whether mitochondrial dysfunction exists in *GLO1*-KO hiPSCs under normal conditions, we performed the transcriptome analysis by RNA-seq (Supplemental materials and method), using previously established WT and *GLO1*-KO hiPSCs^26,27^. A total of 5098 genes were significantly dysregulated (2579 upregulated and 2519 downregulated, *P* < 0.01; Fig. S2A). Molecular pathways for oxidative phosphorylation, eukaryotic initiation factor 2 signaling, and mitochondrial dysfunction were significantly enriched for the downregulated genes as evidenced from the Ingenuity Pathway Analysis (IPA) (Fig. S2B). We also found that the broad downregulation of mRNA encoding oxidative phosphorylation complexes in *GLO1*-KO hiPSCs (Fig. S2C). Given that *GLO1* depletion in hiPSCs elicited enhanced carbonyl stress^26,27^, these results suggest that *GLO1*-KO hiPSCs have mild mitochondrial dysfunction even under normal conditions, which may stem from enhanced carbonyl stress in *GLO1*-KO hiPSCs.

### Enhanced carbonyl stress elicited mitochondrial dysfunction in *GLO1* KO-hiPSC-derived neurons

**Fig. 4:**
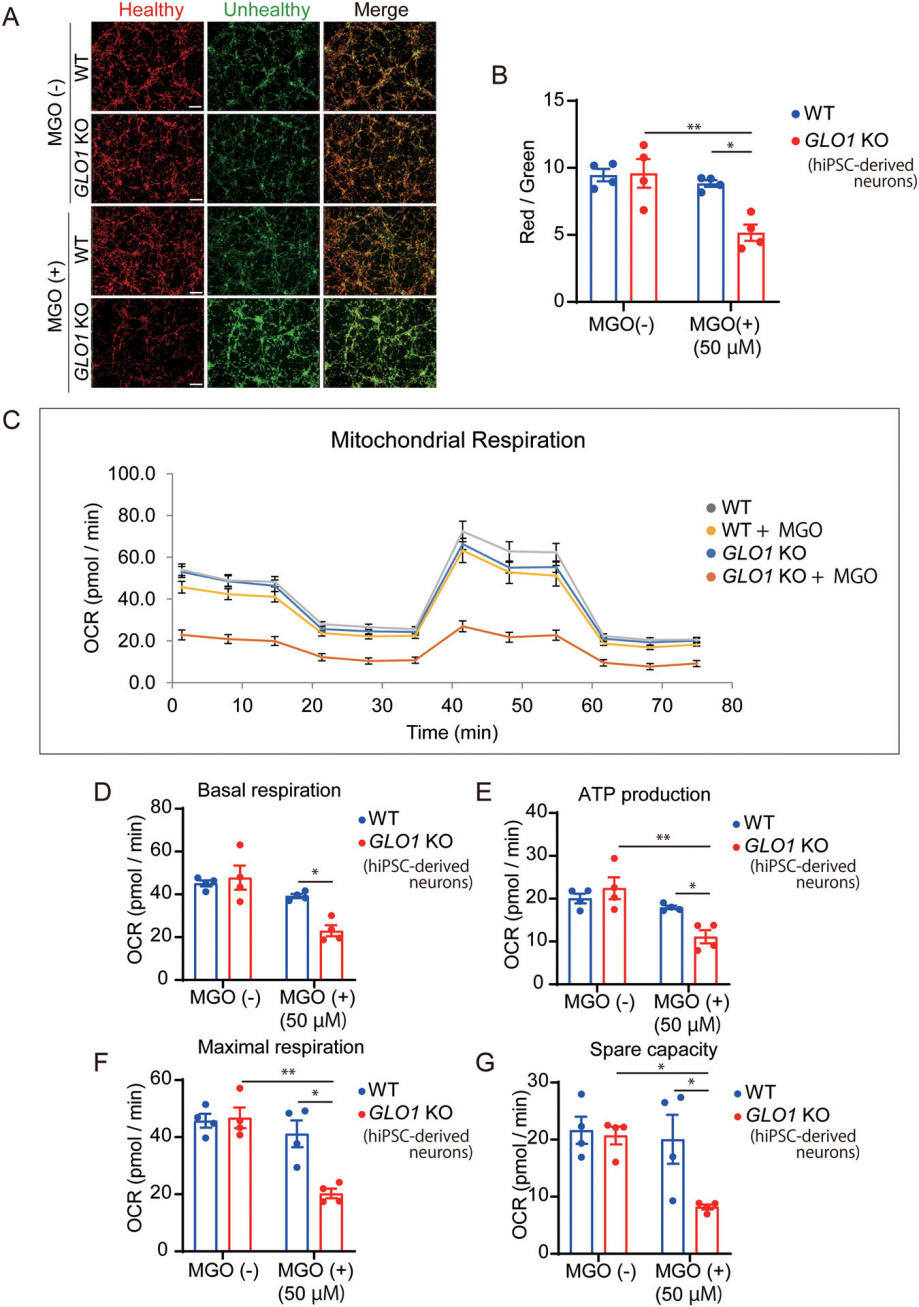
Evaluation of mitochondrial function in WT and *GLO1* KO neurons after treatment with MGO. **A**, **B** JC-1 staining of WT and *GLO1* KO neurons after treatment with 50 µM MGO for 24 h. Scale bar: 100 µm (**A**). The ratio of red to green is an indicator of mitochondrial membrane potential (**B**). **C** Plot of oxygen consumption rate (OCR) in WT and *GLO1* KO neurons using Seahorse extracellular flux analyzer after treatment with 50 µM MGO for 24 h. **D**−**G** Quantification of basal respiration (**D**), ATP production (**E**), maximal respiration (**F**), and spare capacity (**G**). *GLO1* KO neurons showed significantly lower respiratory function after treatment with 50 µM MGO. Data represent mean ± SEM. *P < 0.05, **P < 0.01; two-way ANOVA, followed by Tukey’s multiple comparison test.

As MGO treatment (50 µM, 24 h) caused mitochondrial dysfunction in the *GLO1* KO-hiPSCs, we performed the same measurements in hiPSC-derived neurons. In JC-1 staining, the mitochondrial membrane potential was similar in WT and *GLO1* KO under normal conditions (Fig. 4A, B). However, MGO treatment significantly decreased the mitochondrial membrane potential in the *GLO1* KO-hiPSC-derived neurons (Fig. 4B). In the mitochondrial respiration assay based on the OCR, MGO treatment significantly diminished mitochondrial respiration abilities in the *GLO1* KO-hiPSC-derived neurons (Fig. 4C). Compared to those in the MGO-treated WT hiPSC-derived neurons and/or *GLO1* KO-hiPSC-derived neurons under normal conditions, significant reductions in basal respiration, ATP production, maximal respiration, and spare capacity were observed in the MGO-treated *GLO1* KO-hiPSC-derived neurons (Fig. 4D−G).

### MG-H1, an AGE derived from MGO, accumulated in the mitochondria of *GLO1* KO-hiPSCs and derived neurons under external carbonyl stress

**Fig. 5:**
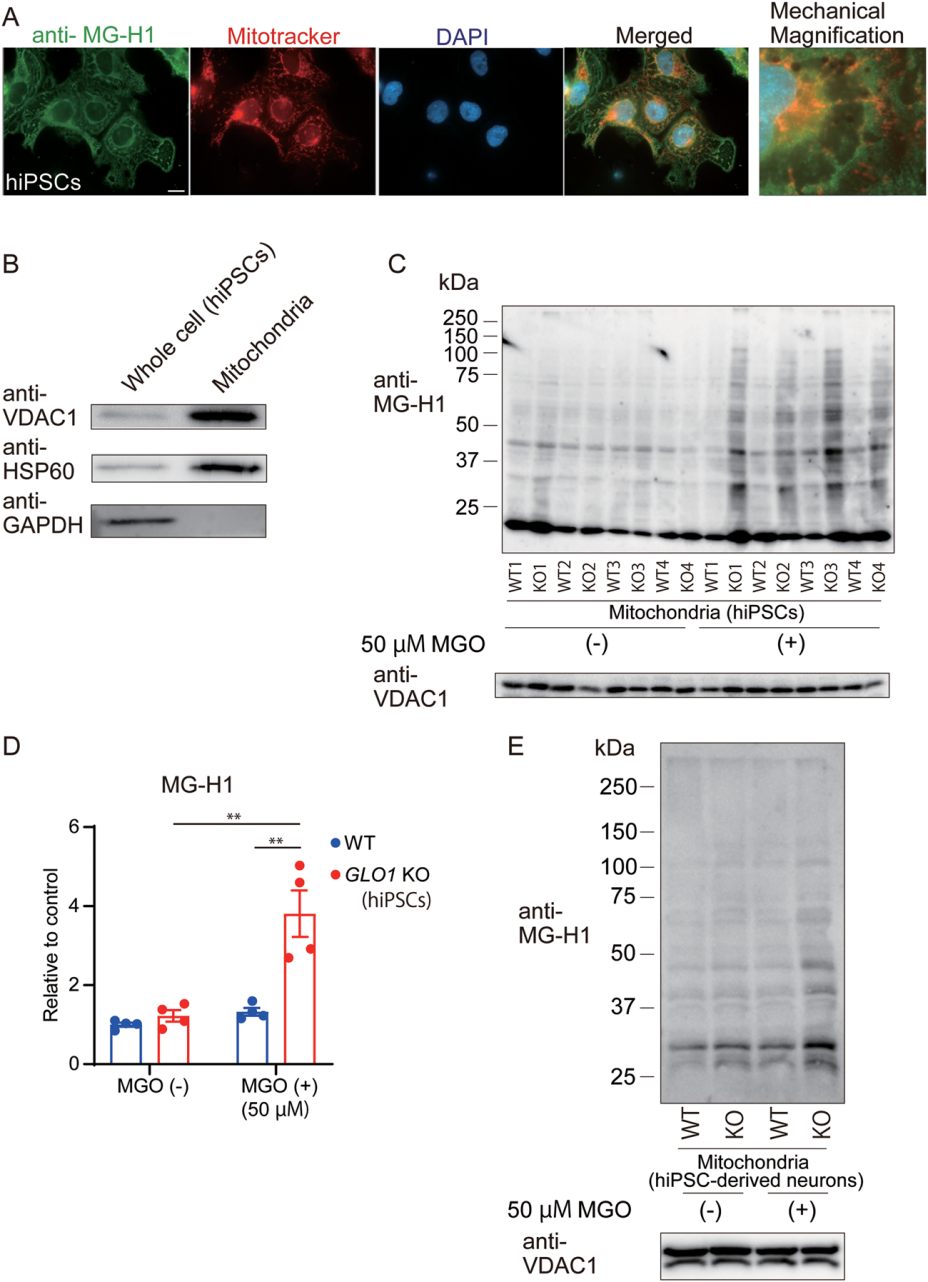
Deposition of MG-H1 and mitochondrial proteins in mitochondria from WT and *GLO1* KO cells. **A** Immunofluorescence images of MG-H1 (green) in mitochondria labeled with Mitotracker^®^ (red) in WT-hiPSCs (scale bar: 20 µm). MG-H1 immuno-positive staining was partially localized in mitochondria. **B** Representative Western blot for VDAC1, HSP60, and GAPDH in lysates from whole cell and isolated mitochondria. **C** Western blotting for MG-H1 and VDAC1 in mitochondria isolated from WT- and *GLO1* KO-hiPSCs after treatment with 50 µM MGO for 72 h. **D** Densitometric analysis of the Western blot shown in **C**. Significant increase in MG-H1 modification was observed in mitochondria from *GLO1* KO-hiPSCs after treatment with 50 µM MGO. **E** Western blotting for MG-H1 and VDAC1 in mitochondria isolated from WT- and *GLO1* KO-derived neurons after treatment with 50 µM MGO for 24 h. Mitochondria from *GLO1* KO-derived neurons showed increase in MG-H1 modification upon MGO exposure. Data represent mean ± SEM. **P < 0.01; two-way ANOVA, followed by Tukey’s multiple comparison test.

To investigate the mechanism of mitochondrial dysfunction, we assessed the accumulation of AGEs elicited by MGO treatment. In the WT hiPSCs, MG-H1 immuno-positive staining co-localized partially with the mitochondrial marker MitoTracker^®^ (Fig. 5A). We further isolated mitochondria from hiPSCs and hiPSC-derived neurons. The purification of isolated mitochondria was confirmed using antibodies against voltage-dependent anion channel 1 (VDAC1) and heat shock protein family D (Hsp60) member 1 (HSP60), both of which are well-established mitochondrial markers (Fig. 5B). Compared to that in non-treated KO and MGO-treated WT-hiPSCs, MG-H1 protein modification increased markedly in the *GLO1* KO-hiPSCs after MGO treatment (50 µM, 24 h), (Fig. 5C, D). Compared to that in the non-treated *GLO1* KO-derived neurons and the WT-derived neurons, increase in protein modification with MG-H1 was also observed in the *GLO1* KO-hiPSC-derived neurons after MGO treatment (Fig. 5E). Note here that the bands for VDAC1 can be single or double depending on experimental conditions^42–44^, which may be due to protein modification(s), alternative splicing, proteolytic breakdown, or other reasons. These data suggested that mitochondrial dysfunction in hiPSCs and derived neurons was partially induced under external carbonyl stress via protein modification with MG-H1.

## Discussion

Accumulating evidence shows the involvement of enhanced carbonyl stress in schizophrenia pathophysiology^5,45,46^. This study showed that hiPSCs and derived neurons harboring deletion in *GLO1*, an important gene responsible for the clearance of carbonyl compounds, elicited biological dysfunction when extrinsic carbonyl stress was induced with MGO treatment. Our results suggested that a certain threshold of carbonyl stress, induced by a combination of genetic defects that increase susceptibility to oxidative stress and external factors, may play a substantial role in schizophrenia pathophysiology.

Reports have shown that MG-H1, an AGE derivative from MGO, accumulates in the mitochondria of *Caenorhabditis elegans* upon disruption of glyoxylase I activity^47^. Therefore, we immunologically evaluated hiPSCs using an antibody against MG-H1. As revealed by perturbed OCRs and accumulation of MG-H1-modified proteins, mitochondrial dysfunction due to carbonyl stress was thought to lead to decrease in cell viability, increase of caspase-3 activity, and dampening of neurite extension and cell migration. Mounting evidence points to the relationship between mitochondrial dysfunction and the pathophysiology of mental disorders^48,49^.

Although not investigated in this study, another possible mechanism of carbonyl stress-mediated cell damage may include the elevation of inflammatory signaling via the receptors for AGEs (RAGE)^11^. MG-H1 has high affinity for RAGE, resulting in the activation of nuclear factor (NF)κB signaling^50^. NFκB increases the release of proinflammatory cytokines such as tumor necrosis factor (TNF)-α, interleukin (IL)−1β, and IL-6, and production of reactive oxygen species^51,52^. These inflammatory signaling pathways lead to mitochondrial impairments, which in turn induce inflammatory responses, yielding a vicious cycle of mitochondrial failure^53^.

hiPSCs and differentiated neurons derived from patients with schizophrenia demonstrated several abnormal phenotypes in vitro, indicating that they can be used for addressing neurodevelopmental abnormalities associated with schizophrenia^35,54^. We observed that MG-H1 immuno-positive staining was co-localized with mitochondria in hiPSCs. This finding prompted us to focus on mitochondrial protein, although other cellular components were also immuno-positive. MGO is a freely membrane permeable compound and reacts with multiple proteins, lipids, and nucleic acids^55^. The multiple mitochondrial proteins from *GLO1* KO-hiPSCs and derived neurons showed increased accumulation of MG-H1 upon MGO stress. MGO is known to modify arginine, lysine, and cysteine residues of various proteins^56^. Rosca et al.^57^ found several protein dots positive for anti-MGO-derived imidazole AGE antibody in two-dimensional electrophoresis using renal mitochondria from diabetic rats. They identified seven mitochondrial electron transport chain proteins from these immunoreactive spots. Future studies are warranted to identify MG-H1-modified proteins in hiPSCs and derived neurons.

Information regarding whether amelioration of carbonyl stress improves schizophrenia symptoms is critical. Pyridoxamine, one of the three forms of vitamin B_6_ (pyridoxine, pyridoxal, and pyridoxamine), is a carbonyl scavenger that inhibits protein glycation under carbonyl stress via metal ion chelation and decomposition of carbonyl intermediates^58^. One open-label trial in which patients with schizophrenia suffered from enhanced carbonyl stress, reported that a pyridoxamine add-on treatment was partially effective^45^. Our findings also showed that pyridoxamine administration could rescue dampened cell viability and apoptosis in *GLO1* KO-hiPSCs under external carbonyl stress. Hence, our data suggested that therapeutic intervention with carbonyl scavengers during neural development provides a beneficial prevention method for those who have multiple inherent risk factors relevant to oxidative stress. In addition, our data indicated that mitochondria may be a therapeutic target for those who have deleterious carbonyl stress burden.

Finally, whether enhanced carbonyl stress is a cause contributing to the development of schizophrenia or is a secondary state-dependent measure following deterioration and/or relapse of the schizophrenic process accompanied by neuroinflammation remains unclear^46,59,60^. We showed here that at least genetic defects in *GLO1* that are reported in schizophrenia^5,61,62^ may result in vulnerability to extrinsic oxidative stress and abnormal neurodevelopment. Further careful analyses are necessary to clarify the causal and mechanistic relationship between carbonyl stress and schizophrenia pathology.

## Supplementary information

Supplemental materials and methods

Supplementary figure S1

Supplementary figure S1
