# Supplementary material for: Glyoxalase I disruption and external carbonyl stress impair mitochondrial function in human induced pluripotent stem cells and derived neurons
Source: Transl Psychiatry. 2021 May 8;11:275. doi: 10.1038/s41398-021-01392-w (PMC8106684; doi:10.1038/s41398-021-01392-w)
Supplement: Supplementary file 1 — Supplemental materials and methods [file 41398_2021_1392_MOESM1_ESM.docx]

**Supplemental materials and methods**

**RNA-seq analysis**

Transcriptome analysis was performed by RNA-seq as described previously^1^. We used another set of WT and *GLO1*-KO hiPSCs established in our previous study (n = 3 for each)^2^. Total RNA was extracted by miRNeasy mini kit (Qiagen; Hilden, North Rhine-Westphalia, Germany). The quality and quantity of RNA were validated by RNA integrity number (RIN) ≥ 9 using Bioanalyzer RNA 6000 Nano Chip (Agilent Technologies; Santa Clara, CA, USA). cDNA library was prepared from 200 ng of total RNA by using TruSeq Stranded mRNA Sample Prep Kit (Illumina; San Diego, CA, USA) following the manufacturer’s instruction. Briefly, poly-A-containing mRNA was purified using polyT oligo-attached magnetic beads. They were heat fragmented and reverse transcribed into first-strand cDNA using reverse transcriptase and random primers. Second-strand cDNA was synthesized by incorporating dUTP followed by the addition of a single “A” nucleotide at the 3’ ends of the blunt fragments for prevention of the ligation with double-stranded cDNA. After adapter ligation (including multiplexing barcodes), the cDNA fragments were amplified by PCR (15 cycles) to create the final cDNA library. The quality, size distribution, and quantity of the cDNA libraries were assessed by 2100 Bioanalyzer (Agilent technologies). The libraries were further sequenced in 100-bp paired-end read format on the HiSeq 2500 platform (Illumina).

The RNA-seq data for the individual samples were de-multiplexed by using the unique index adapters. Quality of the sequence reads were validated by FastQC (https://www.bioinformatics.babraham.ac.uk/projects/fastqc/), and the reads were trimmed for adapter sequences and low-quality bases using the FASTX tool kit (http://hannonlab.cshl.edu/fastx_toolkit/). The reads were further mapped to the human reference genome (GRCh37/hg19, http://hgdownload.cse.ucsc.edu/goldenpath/hg19/chromosomes/) using TopHat with default parameters (v.2.0.14)^3^, utilizing the aligner Bowtie2 (v.2.2.5)^4^. The expression levels were quantified using Cufflinks (v.2.2.1)^3^ based on the read mapping, and were calculated as fragments per kilo base of transcript per million mapped reads (FPKM), corresponding to the UCSC gene annotations for hg19 (http://hgdownload.cse.ucsc.edu/goldenpath/hg19/database/). To test the statistical significance for differential expression among the comparison groups, Student’s *t*-test on log-transformed FPKM values (log_2_ FPKM) was applied. *P* values < 0.01 were considered to be statistically significant and were further analyzed for gene ontology enrichment and pathway analysis.

Visualization of differentially expressed genes using volcano plots was performed in R (<https://www.r-project.org>). Differentially expressed genes were tested for gene ontology enrichment and pathway analysis. Pathway enrichment analysis was performed using the Ingenuity Pathway Analysis (IPA) (Qiagen, content version: 43605602, release date: 2018-03-28). The statistical significance of the enriched canonical signaling pathways was calculated using Fischer’s exact test. *P* values < 0.01 were considered to be statistically significant.

1 Ide, M. *et al.* Excess hydrogen sulfide and polysulfides production underlies a schizophrenia pathophysiology. *EMBO Mol Med* **11**, e10695, doi:10.15252/emmm.201910695 (2019).

2 Toyoshima, M. *et al.* Enhanced carbonyl stress induces irreversible multimerization of CRMP2 in schizophrenia pathogenesis. *Life Sci Alliance* **2**, doi:10.26508/lsa.201900478 (2019).

3 Trapnell, C. *et al.* Differential gene and transcript expression analysis of RNA-seq experiments with TopHat and Cufflinks. *Nature Protocols* **7**, 562-578, doi:10.1038/nprot.2012.016 (2012).

4 Langmead, B., Trapnell, C., Pop, M. & Salzberg, S. L. Ultrafast and memory-efficient alignment of short DNA sequences to the human genome. *Genome Biology* **10**, R25, doi:10.1186/gb-2009-10-3-r25 (2009).

**Figure legends**

**Fig. S1. A graphical representation of the mutation status in *GLO1* KO-hiPSCs.** Single guide RNA (gray color: full length; blue color: PAM recognition sequence) was transduced to hiPSCs to recognize the first exon of *GLO1* (yellow color). The mutation patterns of *GLO1* are illustrated in each allele of four *GLO1* KO-hiPSCs.

**Fig. S2. RNA-seq analysis of WT and *GLO1* KO-hiPSCs.**

(**A**) Volcano plot shows differentially expressed genes in *GLO1* KO-hiPSCs compared to WT (n = 3). Green and blue dashed lines indicate *P*-value thresholds of 0.05 and 0.01, respectively. Top hits among the differentially expressed genes (*P* < 0.01 and absolute fold change > 2) are highlighted in red spots. (**B**) Gene ontology enrichment analysis for the downregulated genes (*P* < 0.01) in *GLO1* KO-hiPSCs in comparison to WT. (**C**) Significantly downregulated genes (*P* < 0.01) were visualized in the canonical pathway for oxidative phosphorylation defined by the IPA.
