# Supplementary figures and images for: Glyoxalase I disruption and external carbonyl stress impair mitochondrial function in human induced pluripotent stem cells and derived neurons
Source: Transl Psychiatry. 2021 May 8;11:275. doi: 10.1038/s41398-021-01392-w (PMC8106684; doi:10.1038/s41398-021-01392-w)

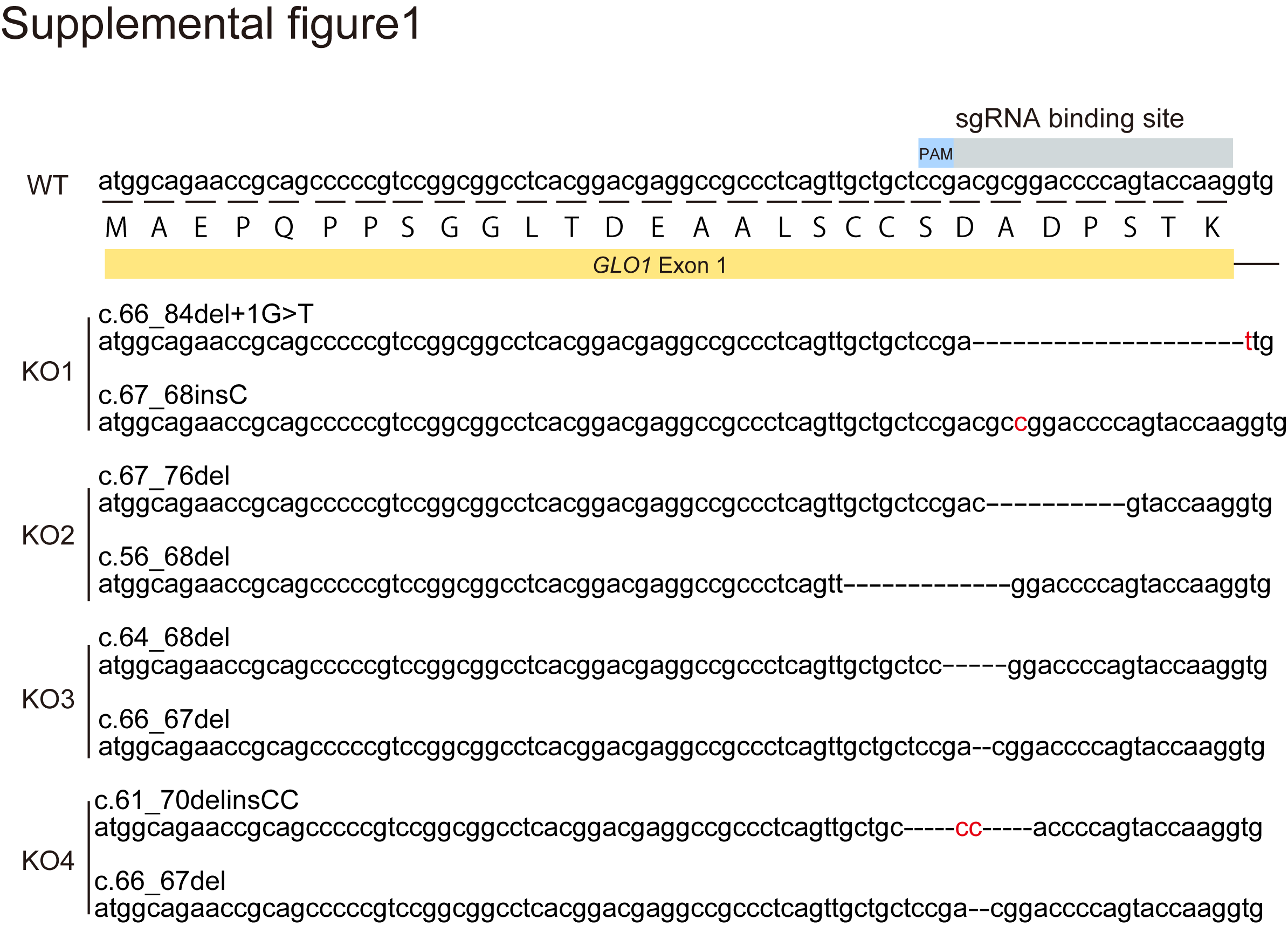

Supplement: Supplementary file 2 — Supplementary figure S1 [file 41398_2021_1392_MOESM2_ESM.tif]

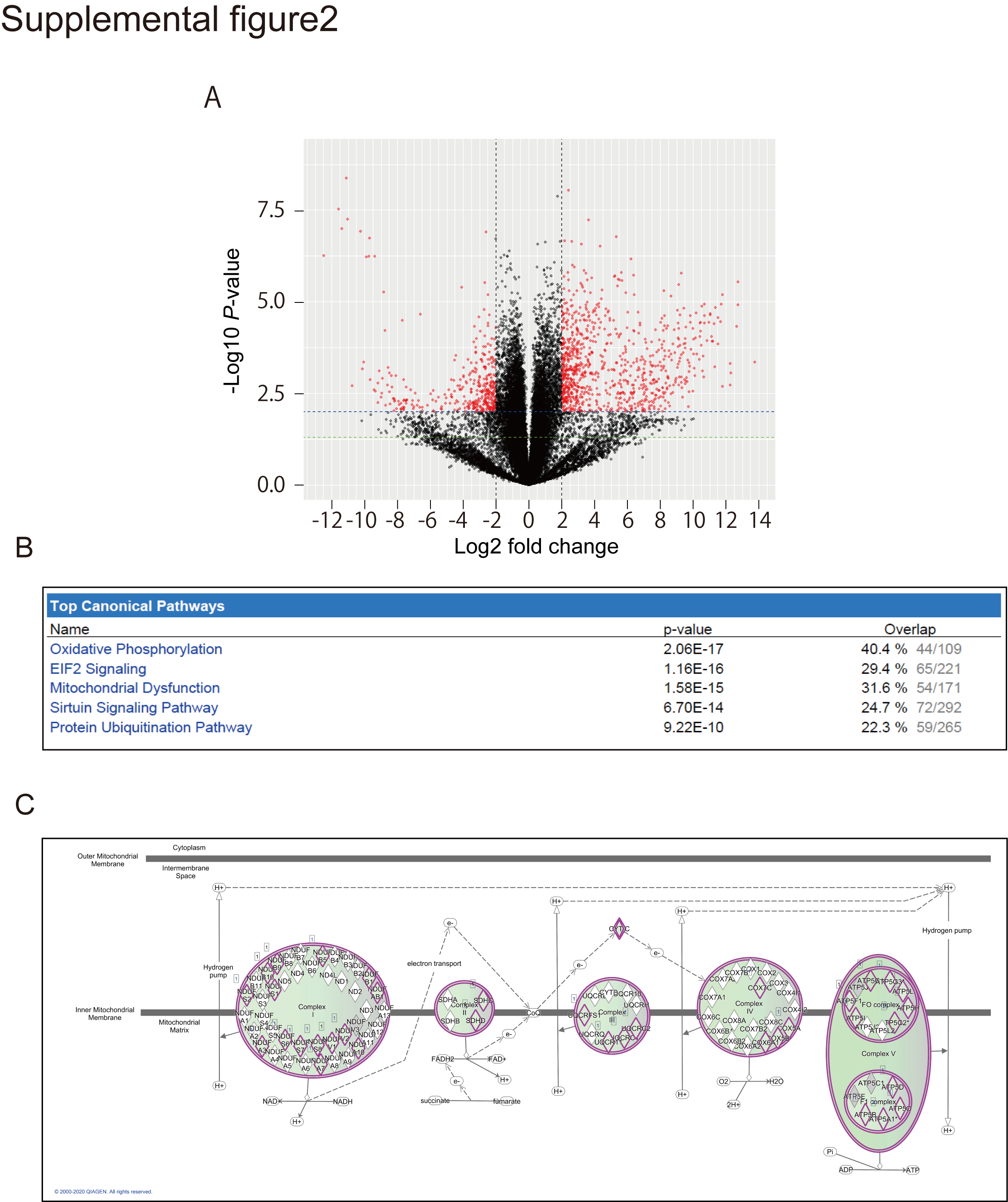

Supplement: Supplementary file 3 — Supplementary figure S1 [file 41398_2021_1392_MOESM3_ESM.tif]
